# Population-centered Risk- and Evidence-based Dental Interprofessional Care Team (PREDICT): study protocol for a randomized controlled trial

**DOI:** 10.1186/s13063-015-0786-y

**Published:** 2015-06-20

**Authors:** Joana Cunha-Cruz, Peter Milgrom, R. Michael Shirtcliff, Howard L. Bailit, Colleen E. Huebner, Douglas Conrad, Sharity Ludwig, Melissa Mitchell, Jeanne Dysert, Gary Allen, JoAnna Scott, Lloyd Mancl

**Affiliations:** Department of Oral Health Sciences, Northwest Center to Reduce Oral Health Disparities, University of Washington, Box 357475, Seattle, WA 98195-7475 USA; Advantage Dental Services, LLC, 442 SW Umatilla Ave, Suite 200, Redmond, OR 97756 USA; Department of Community Medicine and Health Care, UConn Health, 263 Farmington Avenue, Farmington, CT 06030-6325 USA; Department of Health Services, University of Washington, Box 357230, Seattle, WA 98195-7230 USA; Department of Pediatric Dentistry, University of Washington, Box 354915, Seattle, WA 98195-4915 USA

**Keywords:** Dental care, Dental care utilization, Medicaid, Oral health, Children, Pay-for-performance, Randomized controlled trial

## Abstract

**Background:**

To improve the oral health of low-income children, innovations in dental delivery systems are needed, including community-based care, the use of expanded duty auxiliary dental personnel, capitation payments, and global budgets. This paper describes the protocol for PREDICT (Population-centered Risk- and Evidence-based Dental Interprofessional Care Team), an evaluation project to test the effectiveness of new delivery and payment systems for improving dental care and oral health.

**Methods/Design:**

This is a parallel-group cluster randomized controlled trial. Fourteen rural Oregon counties with a publicly insured (Medicaid) population of 82,000 children (0 to 21 years old) and pregnant women served by a managed dental care organization are randomized into test and control counties. In the test intervention (PREDICT), allied dental personnel provide screening and preventive services in community settings and case managers serve as patient navigators to arrange referrals of children who need dentist services. The delivery system intervention is paired with a compensation system for high performance (pay-for-performance) with efficient performance monitoring. PREDICT focuses on the following: 1) identifying eligible children and gaining caregiver consent for services in community settings (for example, schools); 2) providing risk-based preventive and caries stabilization services efficiently at these settings; 3) providing curative care in dental clinics; and 4) incentivizing local delivery teams to meet performance benchmarks. In the control intervention, care is delivered in dental offices without performance incentives. The primary outcome is the prevalence of untreated dental caries. Other outcomes are related to process, structure and cost. Data are collected through patient and staff surveys, clinical examinations, and the review of health and administrative records.

**Discussion:**

If effective, PREDICT is expected to substantially reduce disparities in dental care and oral health. PREDICT can be disseminated to other care organizations as publicly insured clients are increasingly served by large practice organizations.

**Trial registration:**

ClinicalTrials.gov NCT02312921 6 December 2014. The Robert Wood Johnson Foundation and Advantage Dental Services, LLC, are supporting the evaluation.

## Background

Dental caries (tooth decay) predominantly afflict low-income children [1] who also receive fewer dental services [2]. Approximately 45 % of children 3 to 5 years old and fewer than 10 % of children younger than 2 years old received dental services in 2008 in the United States [2]. Many children have their first dental visit in the Emergency Department, and surgical in-hospital treatment of dental caries is becoming more common and costly. Indeed, preventable dental conditions were the primary reason for 830,590 ED visits by Americans in 2009, a 16 % increase from 2006 [3].

Medicaid is the public health insurance program for low-income children in the United States. It is funded by the federal government and states and administered by states. Most Medicaid dental programs operate on a fee-for-service model. These programs largely fail to reduce access disparities because they provide services for those who access care independent of need and because fees are often very low. Where there are managed care systems, they often graft the same limited model with low capitated payments that incentivize less care. In addition, both approaches focus on open-ended budgets versus global payments and office-based versus community-based care. The United States government Centers for Medicaid and Medicare Services expects to move away from the traditional “fee-for-service” system to one based on global budgets, capitation payments, and pay for performance [4]. A recent Cochrane review identified only two studies, both European, on the effects of financial incentives on the delivery of primary dental care [5]. Neither study examined risk-based capitation programs or community-based care systems. Conrad and Perry reviewed the effect of financial incentives on physician behavior and reported that comprehensive financial incentives (for example, balancing rewards and penalties; blending structure, process, and outcome measures; emphasizing continuous, absolute performance standards) offer the prospect of significantly enhancing quality beyond the modest impacts of prevailing pay-for-performance programs [6]. From these reviews, it is also clear that no one has tried to incentivize the entire dental team. Community-based auxiliaries, outreach personnel, case managers, and other practice staff are essential to identify children at risk and to ensure they receive intensive and appropriate management.

### Aims and objectives

To improve the oral health of Medicaid-enrolled children, dental delivery systems innovations are needed, including community-based care, the use of expanded duty auxiliary dental personnel [7, 8], capitation payments, and global budgets [6, 9]. The project aim is to implement and evaluate the PREDICT (*Population-centered Risk- and Evidence-based Dental Interprofessional Care Team*) dental care system. The primary objective is to determine the effectiveness of the new model on decreasing dental caries in Medicaid-enrolled children, pregnant women and new mothers. The secondary objectives are to determine the impact of PREDICT on access, quality, equity and cost.

## Methods/Design

This protocol follows the SPIRIT [10], CONSORT [11] and SQUIRE [12] statements and relevant extensions [13, 14]. The main research question is: Do new delivery and payment systems (compared with the current system) reduce the prevalence of untreated dental caries, and improve access, quality and equity of care for Medicaid-enrolled children, pregnant women and new mothers?

### Design, setting and selection

#### Study design

The study is a cluster parallel-group randomized controlled trial.

#### Setting

The study setting is 14 rural counties in Oregon, USA served by a dental care organization, Advantage Dental Services (ADS), LLC, Redmond, Oregon. ADS is the largest provider of Medicaid dental services in Oregon. It serves about 318,000 members statewide. The organization provides services in 187 primary care and 95 specialist private practices, and 34 staff-model ADS-owned dental clinics. It receives resources from the Oregon Health Authority by capitation payment through regionalized Coordinated Care Organizations (Oregon’s name for Accountable Care Organizations) and has a global budget.

#### Eligibility and recruitment

##### Selection of sites/clusters

In the 14 counties, ADS has a significant Medicaid market share (33 % to 100 %).

##### Selection of participants

The study population is approximately 82,000 children and pregnant women enrolled in Medicaid and living in the 14 counties. The inclusion criteria are as follows:

- Children younger than 21 years of age and women who are pregnant or up to 2 months postpartum.
- Enrollment in Medicaid and assigned to receive dental care from ADS.
- Home address is located in the selected counties.

The age distribution of the children is as follows: 0 to 5 years old, 36 %; 6 to 15 years old, 46 %; and 16 to 20 years old, 17 %. Twenty-one percent of the children and 11 % of the pregnant women are Hispanic, 5 % of children and 4 % of the pregnant women are from other minority races/ethnicities. All pregnant women, new mothers and children <21 years old are enrolled in Medicaid and the Children’s Health Insurance Program.

Recruitment: Participants are identified through a search of the ADS enrollment database. A random subsample of participants is recruited by telephone to participate in an evaluation of the services by completing a telephone survey. Passive informed consent for caries detection and risk assessment at community settings are obtained, as well as active informed consent for treatments provided at these settings.

#### Randomization and blinding

Each County is considered a single cluster. Clusters are randomized with equal probability to the test or control interventions, using computer-generated random numbers. The allocation schedule for random assignment is generated at the University of Washington by the project biostatistician. The treatment allocation for each cluster is kept in Seattle, and the biostatistician informs local investigators about the treatment allocation, when they are ready to conduct the environmental scan of the test sites. Interviewers are blinded to group allocation. Because of the type of the intervention, personnel involved in the delivery of the intervention and participants are not blinded to group allocation.

#### Timing of recruitment, intervention delivery and follow-up

Identification of the clusters (counties) and randomization to test and control interventions was concluded in October 2014. The first phase of the project has been initiated, including the rewriting of job descriptions, identification of appropriate staff members, refinement of outcome metrics, and initial development of IT systems to support the service delivery and incentive payment changes. Formal training of staff will begin in spring 2015; the implementation of the new delivery system will begin in summer 2015; full project implementation will begin when schools open in September 2015 and will last for 24 months. Baseline assessments will be conducted during summer and fall 2015 and final assessments during summer and fall 2017.

### Intervention

#### Development of the program and conceptual framework

**Fig. 1:**
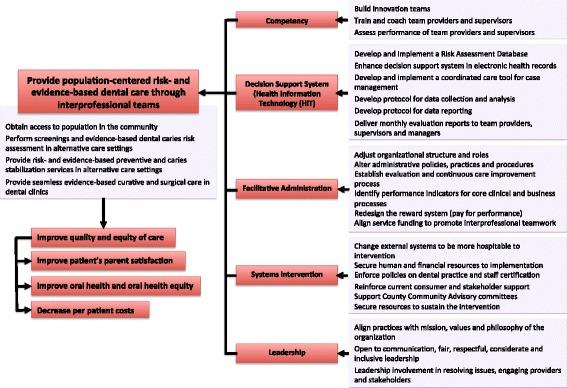
Program logic for PREDICT

We carried out a root cause analysis, based on our previous work on access barriers. We used data from surveys with dental care providers [15–17], pregnant women, new mothers and parents of schoolchildren [18–20] conducted over the past 10 years. Taken together, the major findings include: 1) significant knowledge differences among dentists and hygienists about caring for pregnant women [16] and preschool children [21]; 2) children who had a regular source of care had better oral health [20]; and 3) low-income pregnant women were unaware of Medicaid coverage and/or were uncertain of their acceptance in dental practices [18]. From this work, we created a behavioral model for the use of health services by vulnerable populations [22]. The model posits that disparities are rooted in the health care system and in the social and physical environments of people with fewer resources [23]. In addition to these contextual influences, personal- and family-level factors are important (for example, health beliefs, income, social support, source of care, and skills to navigate the dental system). Considerations of program reach, dental health urgency, cost, organizational readiness, and politics led us to a two-pronged intervention that affects the delivery and the payment systems. The drivers, core components and outcomes of the intervention were operationalized in a multi-level structure based on the Active Implementation Framework [24], and a logic model for the intervention was constructed (Fig. 1). The intervention includes changes at the provider, organization, and system levels and evaluation at the community and patient levels. In addition to program outcomes, we plan to evaluate the extent to which the intervention is delivered as planned.

#### PREDICT - the program

In the seven test counties, Expanded Practice Permit Dental Hygienists (EPPDH) will obtain the consent for treatment at community settings and provide selected primary dental services in non-traditional settings and refer patients to primary care dentists and specialists for complex treatments. Community liaisons and case managers, serving as patient navigators, will help the EPPDH obtain the consents and assure that needed treatments are completed at community and dental office settings. This new delivery system is supported by a financial incentive system for high performance (pay-for-performance, capitation payment and global budget) and by an efficient information system for performance monitoring. The service delivery and payment changes focus on the following: 1) identifying Medicaid-eligible children, pregnant women and new mothers and gaining caregiver consent for services in community settings (for example, schools, Head Start, and WIC Centers); 2) providing risk-based preventive and caries stabilization services efficiently at these settings; 3) providing seamless, effective curative care in dental clinics; and 4) incentivizing local delivery teams to meet performance benchmarks.

#### Control group

The control group consists of seven counties in which Medicaid-eligible low-income children and mothers receive dental care from ADS. This is a “usual care” control with an emphasis on clinic-based care; which includes preventive treatments (not risk based) at some community settings and curative care in dental offices. In the usual care condition the EPPDHs function in a conventional role. Incentives are largely for dentists and not for other team members.

### Study measures and data collection

The primary and secondary study measures for impact and process outcomes are presented in Table 1.

**Table 1:** PREDICT study measures: impact and process outcomes

Type of outcome	Outcome measures	Description	Operationalization
Impact: primary	Oral health	Proportion of children with untreated dental caries	Number of Medicaid ADS-assigned children with untreated caries divided by the number of children enrolled in Medicaid and assigned to ADS.
Impact: secondary	Patient/consumer satisfaction	Mean quality of care	Mean score for the ratings of quality of care from the parent survey
Process: primary	Dental care utilization by children	Percentage of children who received a dental service	Number of Medicaid-enrolled ADS-assigned children who received a dental service (any CDT code) divided by the number of Medicaid-enrolled ADS-assigned.
Process: primary	Consent for treatment at community settings	Proportion of children for whom consent is obtained	Number of Medicaid ADS-assigned children for whom consent is obtained divided by the number of Medicaid ADS-assigned children.
Process: primary	Oral health screening and risk assessment	Proportion of children who received screening and risk assessment	Number of Medicaid-enrolled ADS-assigned children who received both a screening (CDT 0191) and a risk assessment (CDT 0601-D0603) divided by the number of Medicaid-enrolled ADS-assigned children.
Process: primary	Timely need-based curative treatments	Proportion of children referred for dentist treatment who receive care within 60 days of screening	Number of Medicaid-enrolled ADS-assigned children with dentist treatment needs who visited the dentist (CDT D1000 to D9999) within 60 days of screening divided by the number of Medicaid-enrolled ADS-assigned children with dentist treatment needs.
Process: primary	Dental care utilization by pregnant women and new mothers	Percentage of pregnant women and new mothers who received a dental service	Number of Medicaid-enrolled ADS-assigned pregnant women who received a dental service (any CDT code) divided by the number of Medicaid-enrolled ADS-assigned pregnant women and new mothers.
Process: secondary	Risk-based preventive treatments	Proportion of children at high or moderate risk who receive preventive services	Number of Medicaid-enrolled ADS-assigned children considered at high or moderate caries risk (CDT 0602 to D0603) who received any preventive service (CDT 1000 to 1999) divided by the number of Medicaid-enrolled ADS-assigned children considered at high or moderate caries risk (CDT code 0602 to D0603).
Process: secondary	Emergency Department Visit Rate	Percentage of participants who visited an Emergency Department for dental conditions	Number of Medicaid-enrolled ADS-assigned children and women who visited an ED for dental conditions (any CDT code) divided by the number of Medicaid-enrolled ADS-assigned children and women.

#### Structure outcomes

Structure outcomes are identified below:

1. *Readiness for change of the organization:* Organizational Readiness for Implementing Change (ORIC) questionnaire [25].
2. *Team work and communication:* TeamSTEPPS questionnaire [26].
3. *Staff and provider satisfaction:* Work conditions, provider reactions, burnout and quality and safety of care assessed by Minimizing Errors/Maximizing Outcomes (MEMO) questionnaire [27].
4. *Employee Retention and Turnover:* Turnover rate for the members of the team.
5. *Productivity:* Number of procedures for the members of the team.
6. *Health Information Technology (HIT):* Ability for providers with HIT to interchange field and clinic data electronically.

#### Economic outcomes

Economic outcomes include the costs for the following:

1. Outreach activities: actual FTE expenditures on community outreach.
2. Oral health screening and caries risk assessment.
3. Preventive treatment.
4. Restorative treatment.
5. Dental care.
6. Total dental: sum of outreach and dental care costs.
7. Outpatient medical.
8. Emergency department visits.
9. Inpatient hospitalizations.
10. Total medical.
11. Outpatient prescriptions.
12. Total cost: Sum of dental, medical, and prescription costs.

Potential confounders or effect modifiers at the county level include the percentage of participants in different age categories, at elevated caries risk, and in different race/ethnicity categories.

#### Data collection

Data on *impact outcomes* will be obtained through a clinical examination of children and a telephone survey of parents/caregivers and children at baseline and after 24 months. A random sample of Medicaid-enrolled ADS-assigned children will be obtained and stratified by county, at baseline and 24 months post-implementation. The sample will include children whose caregivers either consented or did not consent for care. It will also include children who sought care directly from a dental office. For the survey, staff members will be given a list of enrollees to telephone and move down the list in order until the required sample size is reached. Each individual will be called a minimum of five times. For the clinical examination, staff members will be given a list of enrollees, and they will exam the children in the community settings.

Data on *process and cost outcomes* will be obtained through ADS’s health information systems: Medicaid enrollment database, ADS database (ADIN), electronic dental records and payroll ledger. All Medicaid-enrolled ADS-assigned children population will be included in the process and cost evaluation. Enrollment information including patient identifiers, birth date, gender, race/ ethnicity, coverage dates and assigned primary care provider will be obtained from ADS’s enrollment database and linked to State Medicaid claims. Medicaid claims will be accessed to collect emergency department, medical care and prescription services and costs. Data on *structure outcomes* will be obtained through staff online surveys at baseline and after 24 months and continuously through ADS’s health information systems and internal records. Fidelity information will be obtained through staff surveys and internal records.

### Statistical analysis

#### Sample size

Based on seven test counties and seven control counties, the power is at least 80 % to detect an increase in dental care utilization from pre- to post-implementation within the test counties or an increase in utilization between test and control counties post-implementation of approximately 10 percentage points for utilization rates of 5 % to 10 %, 15 percentage points for utilization rates of 20 % to 30 %, and 20 percentage points for utilization rates of 40 % to 50 %. Estimates are conservative, based on an intracounty correlation (ICC) of 0.05. Pilot data on dental care utilization indicated ICCs were less than 0.05 for cluster sizes of 200 or more children, and ICCs were less than 0.02 for cluster sizes greater than 500 children. Using a sample size of 80 children per county pre- and post-implementation and an ICC of 0.05, we estimate power is 80 % to demonstrate a 15 percentage point or larger decrease in prevalence of untreated dental caries, assuming a prevalence of untreated caries of 25 % in the control counties. In 2012, 25 % of 6 to 10 years old from low-income families in Oregon had untreated dental decay [28]. Using a sample size of 20 parents per county pre- and post-implementation we estimate power is 84 %, based on a two-sided 0.05 significance level, to detect a medium effect of the intervention on dental care ratings (for example, an average increase of 0.75 points) (CAHPS; range 1 to 10), assuming SD = 1.5 in patient satisfaction scores [29].

#### Statistical analysis plan

Descriptive statistics (means, standard deviations, counts, and percentages) are calculated for all variables of interest overall and stratified by age group. The primary hypothesis is that the test intervention participants will have lower number of untreated dental caries than the control participants and the absolute changes indicate a reduction in disparities. Multilevel regression are used to assess for changes in the impact and process outcomes between pre- and post-implementation in the test and control counties, weighted by the number of eligible children/pregnant women in the county at each time point and adjusting for within-county correlation between pre- and post-implementation outcomes [30]. We use multilevel linear regression for mean scores and multilevel binomial regression for binary measures. Additional multilevel regression modeling are used to compare changes in the impact and process outcomes by child age category (for example, 0 to 5, 6 to 15 and 16 to 20 years old) or race/ethnicity, adjusting for clustering within county and weighting by number of eligible children in each age or race/ethnicity category.

#### Economic statistical analysis plan: cost per member per month

The hypotheses are that the new model will increase outreach dental care costs, yet reduce total costs compared with no change in the control model. This is evaluated using a difference in differences approach, adjusting for within cluster variance, to measure the reduction in cost per patient per month for pre versus post intervention effects within the intervention group and for control versus intervention effects. Individual cost components are also evaluated as outcomes in order to elucidate the specific areas of cost which contribute to differences in total costs. Patients in the intervention and control groups are matched using a propensity score based on patient demographics (age, gender, and race), primary medical provider, and primary dental provider. Generalized linear models are constructed using the gamma family with a log-link in order to account for highly skewed cost data. The cost outcomes are the dependent variables while an indicator for the presence of the intervention are the primary independent variable.

#### Cost-effectiveness

The hypothesis is that the new model will be dominant (that is, lower total costs with an increased prevalence of treatment) or highly cost-effective (that is, marginally increased total costs with a significant decrease in the prevalence of untreated caries). We construct an incremental cost-effectiveness ratio with incremental total costs of the PREDICT minus those of the control divided by the difference in the proportion of patients who have untreated caries in PREDICT minus the proportion of patients with untreated caries in the control. We use bootstrapping to estimate a 95 % confidence range for the cost-effectiveness ratio.

### Data management and quality assurance

The dental care organization staff members who have been trained by UW investigators will collect data. Data will be entered in a secure and US Health Information Portability and Accountability Act of 1996-compliant database with range checks for data values. Although we do not expect any adverse events to occur, staff members will be trained to collect, report and manage solicited and spontaneously reported adverse events and other unintended effects of trial interventions or trial conduct. UW investigators do not have access to personal health information and receive only de-identified datasets for analysis.

### Ethics and dissemination

The results from the trial will be published regardless of the outcome. Reporting of this trial will adhere to the relevant and most up-to-date CONSORT [11] and SQUIRE [12] statements and relevant extensions [13, 14]. The investigators ensure that the trial is conducted in compliance with this protocol and federal regulations. The protocol was submitted to and considered by the University of Washington Institutional Review Board, but, consistent with US Federal regulations, participants of this quality improvement project do not meet the criteria to be considered research subjects and ethical approval was deemed unnecessary.

## Discussion

In most states, children enrolled in the Medicaid program have low utilization of dental care and a high prevalence of untreated decayed teeth. Barriers to care and per patient treatment costs can be substantially reduced by a population-based approach to dental care and the provision of basic services outside of traditional clinics. Total expenses increase because more children and mothers receive care. This alternative model does not depend on caregivers taking children to dental offices during work time and can achieve substantial savings in capital costs associated with facilities and equipment and personnel. Children at high risk can be identified and intensive evidence-based effective preventive and curative services provided.

To our knowledge, this intervention will be the first large scale, “real world” test of an alternative delivery and payment model that has the potential to reduce dental care and oral health disparities nationally. This delivery model is replicable in the 47 states where dental hygienists are legally able to provide services to low-income children in community settings under indirect dentist supervision (for example, dentist is not physically present but is on call).

The major challenges for PREDICT are obtaining the permission of community organizations and schools to implement the new dental care system, gaining the cooperation of community leaders, educators, principals and teachers, and getting parents to sign-up for the program. Another challenge is providing the screening, risk assessment and risk-based services to children at community settings cost-effectively. Finally, children with dental diseases or other oral health needs that require treatment in dental offices need to receive it in a timely fashion through well-organized pathways.

Both the evaluation and effective management of this intervention depend on the availability of operational data. First, staff members have to complete annual environmental scans of test and control counties. These scans address differences among counties in availability of dentists, community prevention programs, population income and ethnicity, etc. Second, data are needed on community settings and schools for planning the intervention and evaluation. Third, data are needed on the characteristics and oral health of the children who do/do not consent for the program. Finally, the costs and services provided in test and control dental delivery systems need to be assessed. Determining the categories of cost that are incremental to evaluation versus care delivery (for example, analysis costs and any space, time, and supply costs unique to the evaluation) will also be important. This requires a clear understanding and measurement of all costs, so costs specific to the evaluation can be identified. Realistically, the final cost assignment is best done retrospectively. This is because some evaluation costs may cover business or clinical processes that turn out to be critical for the effective management of the program. As such, they should be considered care delivery costs.

The key to this intervention is effective management and HIT support. Managers and staff members need monthly management reports so they can adjust to problems that arise. ADS has substantial public relations and financial risk, so it is incentivized to make an all-out implementation effort. This “real’ world” operational environment increases the chances for success and the generalizability of the results. Indeed, if the intervention is cost-effective, ADS plans to make it the primary delivery model for Medicaid children and mothers.

The role of financial incentives in the proposed intervention has special importance. Most incentive plans try to influence the behaviors of the dominant providers, in this case, shareholder and employed dentists. However, a careful assessment of the actual intervention indicates that the EPPDHs and administrative staff are the main drivers of program success. This is because only 25 to 30 % of assigned patients will need to be seen by dentists for curative care in the first few years of the program. Moreover, this proportion will decline as the backlog of disease is treated, and new disease is prevented. Developing sensible incentive plans for the nonprofessional staff is not easy, and this will be one of the first studies to address this issue on a large scale in dentistry or medicine. The incentive plan has to take into account the culture of the organization, the interests of the staff in the intervention program, and other factors. For this reason, incentives are mainly positive rather than negative. That is, high performance is rewarded financially, but average or poor performance does not result in substantial reductions to base pay. In the case of below-average performance, emphasis instead is on performance feedback and development of a specific improvement plan.

In summary, we have presented the trial protocol for the PREDICT quality improvement project and discussed the challenges in implementing and evaluating this new dental care delivery and payment systems in a large dental care organization serving Oregon rural counties.

### Trial status

The quality improvement project started in October 2014, and counties have been identified and randomly allocated. Delivery system and payment system changes will be deployed in August 2015 and January 2015, respectively. Data collection for the parent/caregiver/child and staff surveys will be conducted in July and August 2015 and in September and October 2017. The study is not yet recruiting participants.

## Abbreviations

ADS: Advantage dental services
CDT: Code on dental procedures and nomenclature
HIT: Health information technology
